# A secreted schistosome cathepsin B1 cysteine protease and acute schistosome infection induce a transient T helper 17 response

**DOI:** 10.1371/journal.pntd.0007070

**Published:** 2019-01-17

**Authors:** Kateryna Soloviova, Ellen C. Fox, John P. Dalton, Conor R. Caffrey, Stephen J. Davies

**Affiliations:** 1 Department of Microbiology and Immunology, F. Edward Hébert School of Medicine, Uniformed Services University of the Health Sciences, Bethesda, Maryland, United States of America; 2 School of Biological Sciences, Medical Biology Centre, Queen’s University Belfast, Northern Ireland, United Kingdom; 3 Center for Discovery and Innovation in Parasitic Diseases, Skaggs School of Pharmacy and Pharmaceutical Sciences, University of California San Diego, La Jolla, California, United States of America; FIOCRUZ—Minas, BRAZIL

## Abstract

The natural history of schistosome infection in the mammalian host is determined by CD4^+^ T helper responses mounted against different parasite life cycle stages. A T helper 2 (T_H_2) response to schistosome eggs is required for host survival and establishment of chronic infection. However, a T_H_2 cell-derived cytokine also contributes to an immune milieu that is conducive to schistosome growth and development. Thus, the same responses that allow for host survival have been co-opted by schistosomes to facilitate parasite development and transmission, underscoring the significance of CD4^+^ T cell responses to both worms and eggs in the natural history of schistosome infection. Here we show that a cathepsin B1 cysteine protease secreted by schistosome worms not only induces T_H_2 responses, but also T_H_1 and T_H_17 responses, by a mechanism that is dependent on the proteolytic activity of the enzyme. Further investigation revealed that, in addition to the expected T_H_1 and T_H_2 responses, acute schistosome infection also induces a transient T_H_17 response that is rapidly down-regulated at the onset of oviposition. T_H_17 responses are implicated in the development of severe egg-induced pathology. The regulation of worm-induced T_H_17 responses during acute infection could therefore influence the expression of high and low pathology states as infection progresses.

## Author summary

Schistosomiasis, a neglected tropical disease caused by parasites of the genus *Schistosoma*, is prevalent throughout the developing world, with more than 230 million people infected. Left untreated, schistosome infection may cause relatively mild disease with some morbidity, or, in a minority of cases, result in severe pathology and death. These variable outcomes are recapitulated in animal models, where the natural history of schistosome infection is profoundly influenced by the responses of host CD4^+^ T helper cells. Type 2 CD4^+^ T cell (T_H_2) responses, which allow for host survival by limiting pathology, have ironically also been co-opted by schistosomes to promote parasite development. On the other hand, T_H_17 responses have been implicated in the development of severe pathology, in both experimentally infected animals and naturally infected humans. Here we show that a schistosome proteolytic enzyme (SmCB1), produced in the parasite gut and released into the bloodstream, induces both T_H_2 and T_H_17 responses by a mechanism that requires the enzyme’s inherent proteolytic activity. Further investigation revealed that acute schistosome infection also induces a transient T_H_17 response that is rapidly down-regulated once parasite egg-laying commences. Regulation of T_H_17 responses during early infection may help determine whether mild or severe pathology develops as the infection progresses.

## Introduction

Trematodes of the genus *Schistosoma* infect at least 230 million people worldwide, causing hepatointestinal and urogenital schistosomiasis [1]. Schistosomes are the most significant helminthic cause of human morbidity—in 2010, the Institute for Health Metrics and Evaluation’s Global Burden of Disease Study estimated that schistosome infections accounted for over 3.3 million disability-adjusted life years (DALYs) worldwide [2,3]. The relative ease by which schistosome infection is acquired likely contributes to the high global prevalence of schistosomiasis. Unlike other trematodes that infect humans, schistosomes have a truncated life cycle that omits the metacercaria stage, and the cercariae shed by the snail intermediate host can directly infect the definitive mammalian host. Furthermore, the infectious stage does not require ingestion in order to enter the host, instead penetrating the body directly through the skin by secreting proteases that breach the skin’s barrier defenses [4]. Once inside the host, larval schistosomes enter blood vessels, where growth and development into mature adult schistosomes occurs. The developing parasites also use the vasculature to migrate through the body [5], to the vessels that are the preferred final destination for each schistosome species—the mesenteric veins of the hepatic portal system in the case of most species, or the veins draining the urinary bladder in the case of *S*. *haematobium*, the etiologic agent of urogenital schistosomiasis. In these vessels adjacent to mucosal sites, adult schistosomes produce eggs that traverse the mucosal tissues and return to the environment, continuing the life cycle and disseminating the infection to new hosts.

The natural history of schistosome infection in the mammalian host is determined by host immune responses mounted against different parasite life cycle stages. A CD4^+^ T helper 2 (T_H_2) response to schistosome eggs deposited in host tissues is required for host survival and establishment of chronic infection, as this response mediates formation of protective granulomas around parasite eggs [6,7], while also limiting detrimental pro-inflammatory processes [8–10]. Furthermore, this T_H_2-mediated granulomatous reaction is also required for passage of the eggs through mucosal tissues and their release into the environment [11,12], a necessary event in life cycle propagation.

In mouse models of schistosome infection, CD4^+^ T cells also play an enigmatic role in the development of schistosomes during the pre-patent period of infection, i.e., prior to the onset of oviposition [13,14]. In the specific absence of CD4^+^ T cells, developing schistosomes exhibit reduced growth rates, delayed reproductive maturation, and impaired reproductive fitness [13,15]. Precisely how CD4^+^ T cells influence schistosome development is not clear, but our data suggest a role for CD4^+^ T cell-derived cytokines in contributing to an immune milieu that is conducive to schistosome development [16]. While important roles for other cytokines cannot be excluded, the principal T_H_2 cytokine interleukin (IL-) 4 can, by itself, restore parasite development and reproduction in the absence of CD4^+^ T cells by a mechanism that, again, involves down-regulating pro-inflammatory signals [17]. T_H_2 responses, therefore, play a significant dual role in the natural history of schistosome infection. Whereas T_H_2 responses allow host survival in the face of schistosome egg-induced inflammation, these same responses have been co-opted by schistosomes to promote parasite development and transmission to new hosts.

Schistosome eggs are the principal inducers of T_H_2 responses during schistosome infection [18,19]. However, the possible contribution of T_H_2 cells to facilitating parasite development during pre-patent infection [17] led us to investigate how these responses are induced prior to the onset of oviposition. We previously showed that infection with schistosome worms alone was sufficient to induce systemic, antigen-specific T_H_2 responses that were accompanied by production of antigen-specific immunoglobulin E (IgE) and sensitization of circulating basophils to produce additional IL-4 in response to schistosome worm antigens [20]. We also demonstrated that a schistosome cysteine protease, *S*. *mansoni* cathepsin B1 (SmCB1), which is secreted from the gut of the parasite, was a principal target of the IgE response [21], suggesting this worm-secreted antigen may contribute significantly to T_H_2 polarization of the nascent CD4^+^ T cell response during early infection.

Cysteine proteases possess T_H_2-polarizing properties and may be key T_H_2-inducing components of many helminths and allergens [22,23]. One hypothesis for the immunostimulatory properties of cysteine proteases stems from the observation that vertebrate hosts typically maintain cysteine proteases under tight control in intracellular compartments and do not release these enzymes into the extracellular space [24]. In contrast, many helminths utilize cysteine proteases in critical processes such as host invasion and nutrient acquisition and, as a consequence of these activities, release cysteine proteases in large quantities into the extracellular environment [25,26]. The vertebrate immune system may, therefore, have evolved mechanisms to detect extracellular protease activity and to interpret this as a danger signal associated with helminth infection [27]. The mechanism by which the host immune system senses and responds to foreign protease activity has not been fully elucidated. Protease-activated receptors (PARs) have been proposed to serve as receptors for extracellular protease activity [28–30]. PAR activation can stimulate cells to exocytose adenosine triphosphate (ATP) [31], which then acts as an endogenous danger signal by activating the P2Y_2_ purinergic receptor and stimulating release of IL-33, a T_H_2-promoting alarmin [32]. Alternatively, proteases may damage host cells directly, causing release of IL-33, thymic stromal lymphopoietin (TSLP) and IL-25, which together promote T_H_2 cell differentiation by stimulating type 2 cytokine release by innate lymphoid type 2 cells (ILC2s) (reviewed in [33]).

In addition to the potential role of SmCB1 in the natural history of schistosome infection, this protease is also of interest as a novel anti-schistosome vaccine candidate [34,35]. When administered to laboratory rodents in the absence of adjuvant, either alone or in combination with other antigens, active SmCB1 confers significant protection against challenge infection [36,37], which is dependent on SmCB1 proteolytic activity [37], underlining the innate adjuvanticity of active SmCB1. We therefore sought to test the contribution of SmCB1 activity to CD4^+^ T cell induction during natural infection and to test the immunostimulatory properties of active SmCB1 directly. Our results provide fresh insights into the immune responses induced by SmCB1 and by schistosome worms during pre-patent infection, and may have relevance for understanding the pathogenesis of severe pathology later in infection.

## Materials and methods

### Ethics statement

All animal procedures were performed according to the current edition of the National Research Council’s *Guide for the Care and Use of Laboratory Animals* (The National Academies Press, 2011) and pre-approved by the Institutional Animal Care and Use Committee at the Uniformed Services University of the Health Sciences, permit number A3448-01. Prior to sample collection, all mice were euthanized with an overdose of pentobarbital, or by carbon dioxide inhalation, followed by cervical dislocation or bilateral thoracotomy, in accordance with guidelines issued by the American Veterinary Medical Association’s Panel on Euthanasia.

### Mice, parasite infection and antigen preparation

C.129-Il4^tm1Lky^/J (4get; BALB/c genetic background) breeding pairs were purchased from The Jackson Laboratory (Bar Harbor, ME) and bred at the Uniformed Services University Central Animal Facility to produce offspring for experiments. Wild type 6–8 week old BALB/cJ and C57BL/6J mice were purchased from The Jackson Laboratory. Age- and sex-matched mice (4–5 animals per group) were infected percutaneously by immersion of the tail for 30 min in water containing 120–160 *S*. *mansoni* cercariae (NMRI strain) shed from infected *Biomphalaria glabrata* (NMRI strain) snails. At 4, 6 or 8 weeks post infection, mice were euthanized and blood, spleen and lymph nodes harvested for analysis. In some experiments, worms were perfused from the portal circulation, fixed in 4% neutral buffered formaldehyde, counted, imaged and measured using ImageJ software (https://imagej.nih.gov/ij/). For preparation of soluble schistosome worm antigen (SWAP), adult *S*. *mansoni* were perfused from the portal veins of infected mice and homogenized in sterile phosphate buffered saline on ice. Insoluble material was removed by centrifugation at 16,100 x *g* for 30 min at 4 °C and the resulting supernatant stored at -80 °C until use, after filter sterilization through 0.2 μm syringe filters and determination of protein concentration by Bradford assay.

### Protease inhibitor treatment

Beginning on the day of infection, the cysteine protease inhibitor K11777 (N-methylpiperazine-phenylalanyl-homophenylalanyl-vinylsulfone-phenyl, solubilized in sterile distilled water at a concentration of 10 mg/ml) was administered once daily (SID) by intraperitoneal (IP) injection in volumes of 20 μl/mouse, at a dose of approximately 10 mg/kg [38]. Control mice received equal volumes of vehicle (water) alone. Daily administration was continued until the mice were euthanized at 4 weeks post infection.

### SmCB1 immunization

Active recombinant *S*. *mansoni* cathepsin B1 (SmCB1) was expressed in *Pichia pastoris* and purified by NTA-affinity chromatography as previously described [39,40]. Prior to immunization, batches of SmCB1 were divided in roughly equal portions of approximately 1 mg. One portion was inactivated by incubation with 10 μM E-64 on ice for 1 h. The inactivated portion and the remaining active portion were then desalted into sterile 0.1 M phosphate buffer, pH 5.5, using PD SpinTrap G-25 columns (GE Healthcare Life Sciences) and protein concentration checked by the BCA Protein Assay (Thermo Fisher Scientific). Endotoxin was not detectable in protease preparations using a *Limulus* amebocyte lysate assay (Lonza Inc). No protease activity was detectable in the E-64-inactivated enzyme using the fluorogenic peptidyl substrate, Z-Phe-Arg-7-amino-4-methylcoumarin (Z-FR-AMC, Sigma Aldrich Chemical Co.), as previously described [41]. Active and inactivated enzyme was administered to separate groups of mice on day 0, 14 and 21 by (i) subcutaneous (SC) injection into the footpad (10 μg/mouse), (ii) by IP injection (25 μg/mouse), or (iii) by intravenous (IV) injection into a lateral tail vein (25 μg/mouse). Twenty-four hours after the third injection, spleens and mesenteric, popliteal and inguinal lymph nodes were collected for analysis.

### Quantification of antigen-specific IgE

Plasma concentrations of SWAP-specific IgE were determined as described previously [21]. Briefly, Immulon 4HBX plates (Thermo Fisher Scientific) were coated with SWAP in borate buffered saline (BBS) for 2 h at room temperature, then blocked with BBS containing 1% fetal bovine serum (FBS). Plasma samples were first adsorbed by incubating with GammaBind G Sepharose (GE Healthcare Life Sciences) overnight at 4°C, to deplete antigen-specific IgG that might out-compete the IgE for antigen binding, then applied to the assay plates in serial dilutions in BBS containing 0.02% Tween-20 (Sigma). After washes and incubation with alkaline phosphatase-conjugated goat anti-mouse IgE (BD Biosciences), the reaction was developed by addition of p-nitrophenyl phosphate disodium salt substrate (PNPP, Thermo Fisher Scientific), stopped with 2 N NaOH and the absorbance measured at 405 nm.

### Flow cytometric analysis

Single cell suspensions of spleen and lymph node cells were first incubated with anti-murine Fcγ receptor II/III monoclonal antibody (mAb) 2.4G2 for 10 min and then stained with saturating concentrations of Alexa Fluor 488-conjugated, allophycocyanin-conjugated, PE-conjugated, FITC-conjugated, PerCPCy5.5-conjugated, Alexa Fluor 700-conjugated and Pacific Blue-conjugated mAbs to CD3, CD4, CD8, and CD62L, purchased from either BD Biosciences (San Jose, CA), BioLegend (San Diego, CA), eBioscience (San Diego, CA), or Invitrogen (Carlsbad, CA). *Ex vivo* intracellular staining for IFN-γ, IL-4, GATA-3, IL-17A, and RORγt was performed using antibodies and reagents purchased from BD Biosciences (San Jose, CA) or Biolegend (San Diego, CA) and staining performed according to the manufacturer’s instructions. A full list of antibodies used is provided in S1 Table. Following completion of the staining protocol, cells were analyzed immediately using a BD LSRII flow cytometer (BD Biosciences, San Jose, CA). Lymphocytes were gated by forward and side scatter, and fluorescence data were collected for a minimum of 10,000 gated cells. A representative gating strategy is outlined in S1 Fig

### *In vitro* restimulation of T cells with antigen

Spleen and lymph node cells were washed, resuspended in medium, and plated at 10 × 10^6^ cells (splenocytes) or 2 × 10^6^ cells (lymph node cells) per well in 96-well plates. All cells were cultured in complete medium which consisted of RPMI-1640 supplemented with 10% fetal bovine serum, 2 mM L-glutamine, 50 μM 2- mercaptoethanol, 100 U/mL penicillin and 100 μg/mL streptomycin. For antigen stimulation, SWAP was added to a final concentration of 50 μg/ml. Cells were incubated at 37 °C in a humidified atmosphere containing 5% CO_2_ and were monitored daily. Cells and supernatant were harvested for analysis after 72 hours.

### IFN-γ and IL-17A ELISA

Cytokine concentrations in cell culture supernatants were measured by sandwich enzyme linked immunoassay (ELISA), using ELISA kits from Invitrogen. Assays were performed according the manufacturer’s protocols. Briefly, Costar 9018 ELISA plates were coated with capture antibody overnight at 37°C and then blocked with ELISA/ELISASPOT Diluent. Supernatant (100 μl) was added to the blocked wells and incubated for 2 h at room temperature. After washing, 100 μl/well of detection antibody was applied for 1 h at room temperature. Avidin-horseradish peroxidase (HRP) was applied for 30 min after extensive washing. Finally, 3,3',5,5'-Tetramethylbenzidine (TMB) solution was added for 30 min after washing, the reaction was stopped by Stop Solution (Invitrogen) and absorbance was measured with a Spectramax M2 plate reader (Molecular Devices).

### Cytokine expression by real time PCR

Splenocytes (10 x 10^6^) and cells from mesenteric lymph nodes (5 x 10^6^) were homogenized in 1 ml of RNA-STAT-60 (Tel-Test, Friendswood, TX). cDNA was synthesized from mRNA using the TaqMan Reverse Transcription Reagents kit (Applied Biosystems, Foster City, CA). Real-time PCR was performed using TaqMan Gene Expression Assays and TaqMan Universal PCR Master Mix (Applied Biosystems) for the following targets: IFN-γ, IL-17A and IL-4, with 18s rRNA as an internal control. The calculation of relative gene expression differences was done by the comparative 2^-ΔΔCT^ method [42]. The result was expressed as the -fold change in the experimental groups compared to equivalent cells from non-infected control mice.

### Statistical analysis

Statistical analyses were performed using Prism 7.0 (GraphPad Software, San Diego, CA). F tests were used to compare variances between two data groups, and Brown-Forsythe and Bartlett’s tests were used to compare standard deviations in experiments with three data groups. Where parametric tests were appropriate, the means of two data groups were compared using unpaired *t* tests, and the means of three groups were compared by one-way ANOVA followed by Tukey’s multiple comparisons test. Where non-parametric tests were required, the means of two data groups were compared using the Mann Whitney test, and the means of three groups were compared using the Kruskal-Wallis test followed by Dunn’s multiple comparisons test. *P* values < 0.05 were considered significant. Experimental groups comprised 4–5 mice and all experiments were performed at least twice.

## Results

### *In vivo* cysteine protease inhibition impairs CD4^+^ T cell responses induced by acute schistosome infection

**Fig 1:**
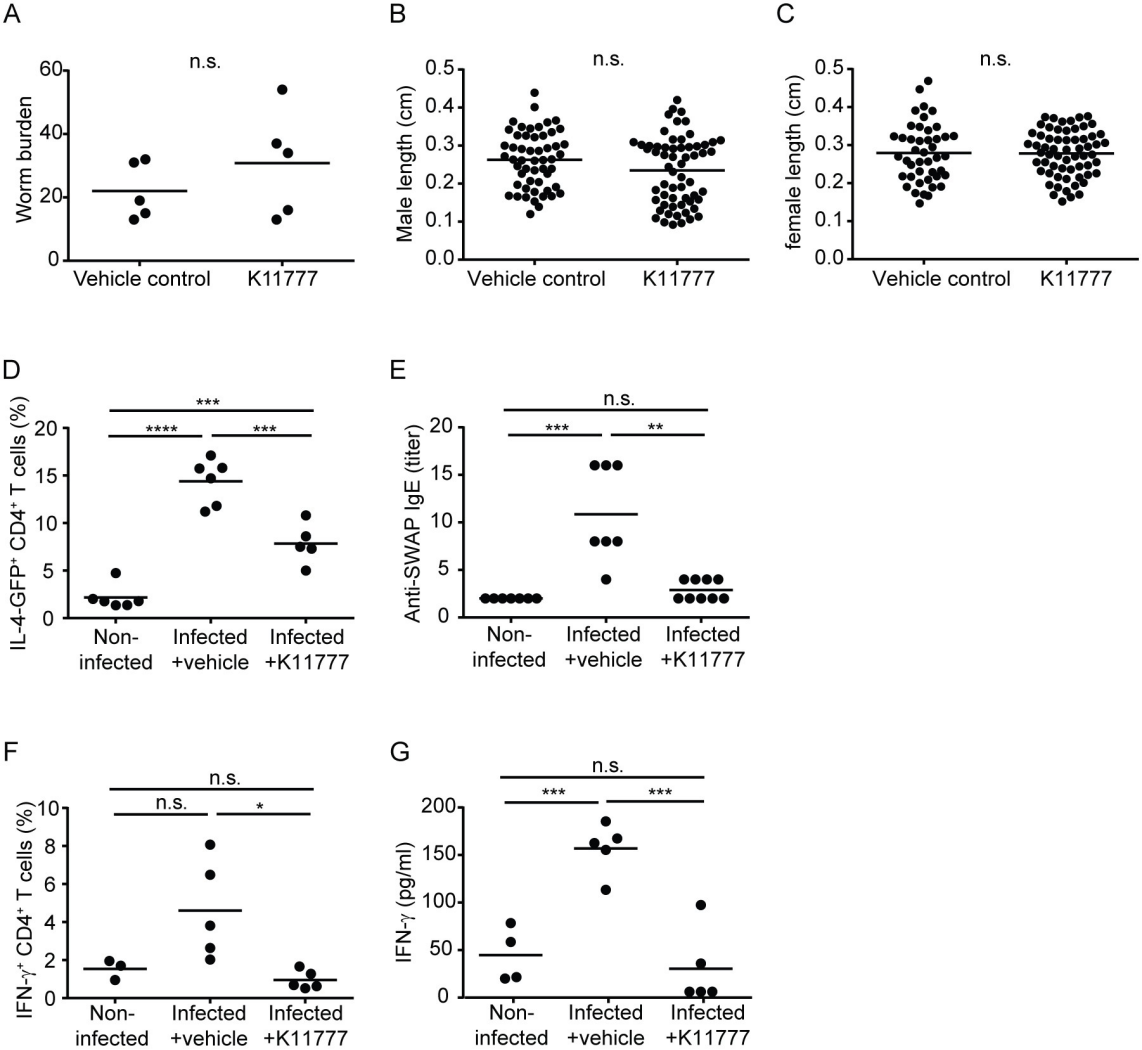
Treatment of mice with the cysteine protease inhibitor, K11777, inhibits T helper responses during pre-patent schistosome infection. *S*. *mansoni*-infected 4get IL-4 reporter mice were treated with cysteine protease inhibitor (K11777) or vehicle alone throughout the first 4 weeks of infection. A, parasite burden, B length of male worms, and C, length of female worms in animals treated with K11777 or vehicle alone, at 4 weeks post infection. D, frequency of IL-4-GFP^+^ CD4^+^ T cells in spleens of non-infected control mice, and vehicle-treated and K11777-treated infected mice at 4 weeks post infection. E, Plasma titers of anti-SWAP IgE in non-infected control mice, and vehicle-treated and K11777-treated infected mice at 4 weeks post infection. F, frequency of IFN-γ^+^ CD4^+^ T cells in spleens of non-infected control mice, and vehicle-treated and K11777-treated infected mice at 4 weeks post infection. G, SWAP-stimulated IFN-γ secretion by splenocytes from non-infected control mice, and vehicle-treated and K11777-treated infected mice at 4 weeks post infection. n.s., *P* > 0.05; *, *P* < 0.05; **, *P* < 0.01; ***, *P* < 0.001; ****, *P* < <0.0001.

To test whether the catalytic activity of cysteine proteases was necessary to the induction of T_H_2 responses during pre-patent schistosome infection, we treated infected IL-4-eGFP reporter (4get) mice with the cysteine protease inhibitor, K11777, beginning on the day of infection and continuing daily for four weeks. Previous studies showed that this vinyl sulfone is well tolerated by schistosome-infected mice and decreases the amount of active SmCB1 detectable in those parasites recovered post-treatment [38]. At higher doses (25–50 mg/kg twice daily [BID]), K11777 reduced worm and egg burdens in infected mice [38]. For the purpose of these experiments, we used a lower dose (10 mg/kg SID) that did not interfere with schistosome establishment or growth in the mammalian host. In our studies, K11777-treated mice harbored similar worm burdens to vehicle control animals (Fig 1A) and the male and female worms from K11777-treated mice were the same size as those from control animals (Fig 1B and 1C). Consistent with a role for SmCB1 activity in driving T_H_2 responses in mice, K11777 treatment reduced the frequency of eGFP^+^ CD4^+^ T cells in the spleens of infected mice at 4 weeks post infection (Fig 1D), and reduced the titers of worm-specific IgE in the plasma of infected mice (Fig 1E). However, the inhibitory effect of K11777 was not specific to T_H_2 responses. Inhibitor-treated mice also exhibited reduced frequencies of splenic IFN-γ^+^ CD4^+^ T cells (Fig 1F), and their splenocytes produced less IFN-γ when cultured with parasite antigen *in vitro* (Fig 1G). These findings suggested that SmCB1 is involved in the induction of other T_H_ phenotypes, in addition to T_H_2. However, we could not rule out the possibility that K11777 was modulating immune responses via off-target effects, e.g. by inhibiting other parasite proteases, by interfering with parasite development (and the subsequent immune response) in aspects that were not apparent from our analysis of parasite morphology, or by interfering with host proteases. To address these issues, we next examined the immunological role of SmCB1 proteolytic activity by comparing immune responses to active and inactive recombinant SmCB1.

### Induction of T_H_2 cells by SmCB1 requires its endogenous proteolytic activity

**Fig 2:**
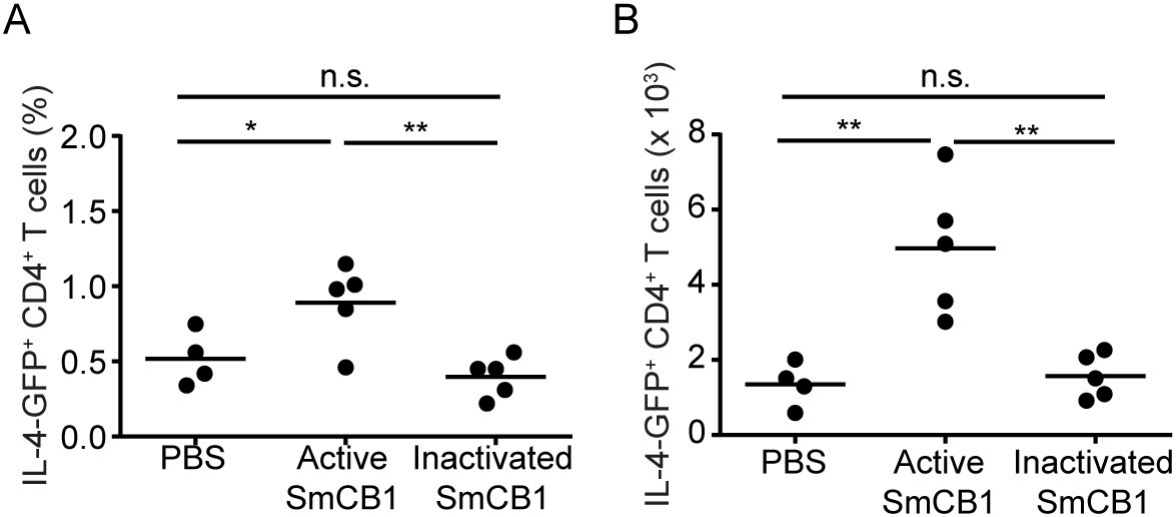
SmCB1 proteolytic activity induces IL-4 expression in CD4^+^ T cells. 4get IL-4 reporter mice were immunized subcutaneously in the footpad with either PBS, active SmCB1, or inactivated SmCB1 on day 0, 14 and 21. On day 22, the draining popliteal and inguinal nodes were removed and the frequency (A) and total number (B) of IL-4-GFP^+^ CD4^+^ T cells were determined by flow cytometry. n.s., *P* > 0.05; *, *P* < 0.05; **, *P* < 0.01.

To examine whether the proteolytic activity inherent to SmCB1 was necessary to induce T_H_2 cells, IL-4-eGFP reporter mice were injected in the hind footpad with either active or E-64-inactivated recombinant SmCB1, and the accumulation of eGFP^+^ cells in the draining popliteal and inguinal lymph nodes was compared. Immunization with active SmCB1 resulted in modest but significant increases in the frequency (Fig 2A) and absolute number (Fig 2B) of T_H_2 cells in the draining nodes. In contrast, immunization with inactivated SmCB1 had no effect, comparable to mice injected with PBS alone (Fig 2A and 2B). However, the low yield of cells from these nodes precluded the analysis of other T_H_ phenotypes that might be present, preventing us from testing whether SmCB1 is involved in the induction of other T_H_ phenotypes, in addition to T_H_2, as suggested by the results obtained with *in vivo* administration of K11777 (Fig 1). We therefore sought to immunize mice by other routes to allow for the collection of larger numbers of responding cells, and to use conventional intracellular cytokine and transcription factor staining, so that multiple T_H_ phenotypes could be examined simultaneously.

### Active SmCB1 induces T_H_1, T_H_2 and T_H_17 cells

**Fig 3:**
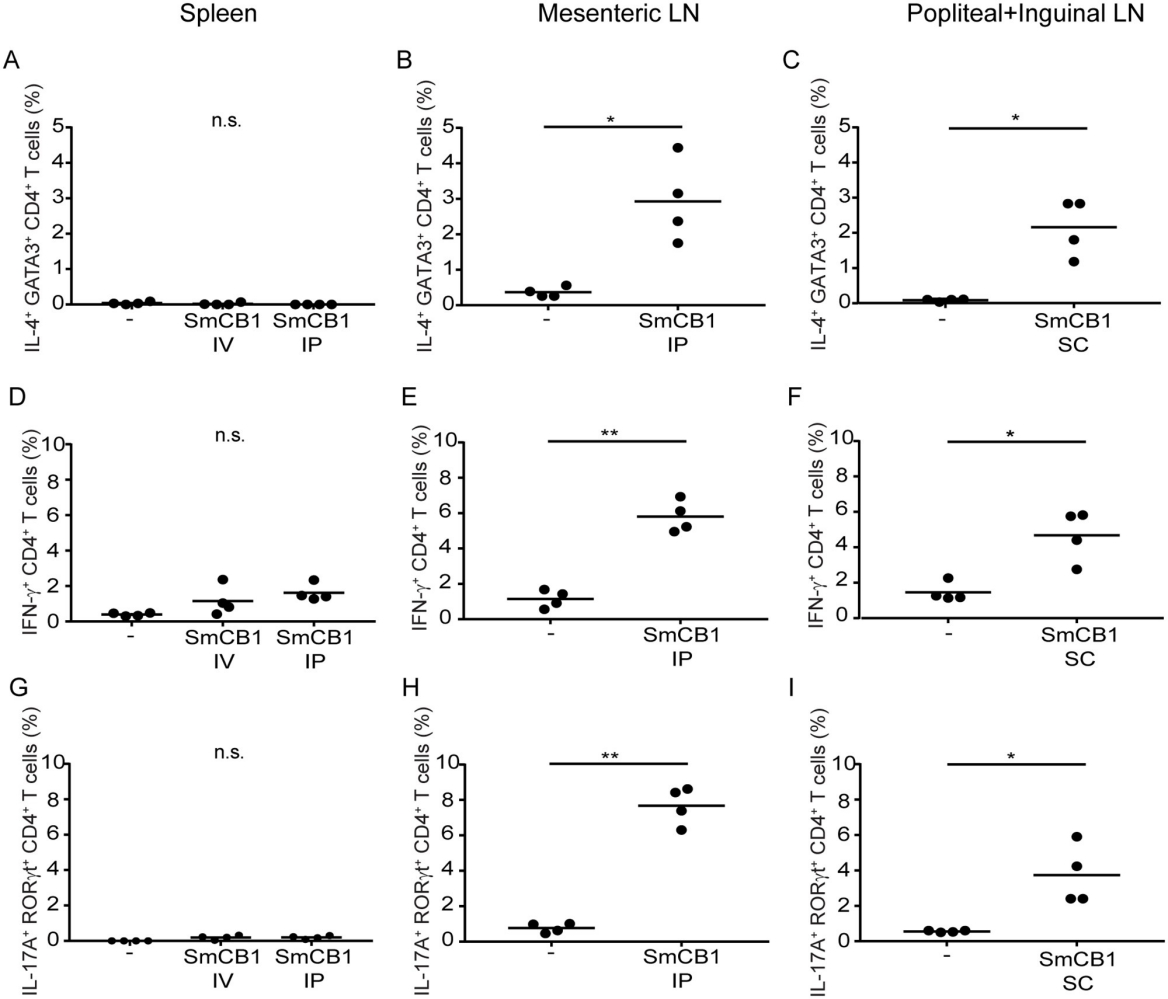
Active SmCB1 induces T_H_2, T_H_1 and T_H_17 cells when administered by intraperitoneal and subcutaneous routes. On day 22, intracellular cytokine/transcription factor staining and flow cytometry were used to determine the frequencies of IL4^+^ GATA3^+^ CD4^+^ T cells (A-C), IFN-γ^+^ CD4^+^ T cells (D-F), and IL-17A^+^ RORγt^+^ CD4^+^ T cells (G-I), in the spleens (A, D, G), mesenteric nodes (B, E, H), and popliteal and inguinal nodes (C, F, I) of wild type C57BL/6 mice that received injections of active SmCB1 on days 0, 14 and 21, by intravenous (IV; A, D, G), intraperitoneal (IP; A-B, D-E, G-H) and subcutaneous (SC; C, F, I) routes. Frequencies were compared to those in the comparable tissues of animals that received no antigen. n.s., *P* > 0.05; *, *P* < 0.05; **, *P* < 0.01.

So that our data would be directly comparable to our previous studies on immune responses to SmCB1 [21], and to test whether SmCB1-induced responses were influenced by the genetic background of the mice used, further immunization experiments were conducted with wild type C57BL/6J mice (the 4get mice utilized in Figs 1 and 2 have a BALB/c genetic background). To evaluate the effect of active SmCB1 when administered by different routes in these mice, SmCB1 was administered to separate groups of C57BL/6J mice on day 0, 14 and 21 by (i) IV injection into a lateral tail vein (25 μg/mouse), or (ii) by IP injection (25 μg/mouse), or (iii) SC injection into the footpad (10 μg/mouse; identical to the route of administration employed in Fig 2). On day 22 (the day after the third injection), the mice were euthanized and flow cytometry was used to compare the T_H_ cell frequencies in the draining popliteal and inguinal nodes of the SC group with the frequencies in the spleens of the IV and IP groups and the mesenteric lymph nodes of the IP group. T_H_2 cells were defined as IL-4^+^ GATA3^+^ CD4^+^ T cells, T_H_1 cells were defined as IFN-γ^+^ CD4^+^ T cells, and T_H_17 cells were defined as IL-17A^+^ RORγt^+^ CD4^+^ T cells. Representative fluorescence histograms are provided in S2 Fig Neither IV nor IP administration of active SmCB1 induced any increase in the frequency of T_H_2 cells in the spleen (Fig 3A). In contrast, IP administration of active SmCB1 induced a significant increase in T_H_2 cell frequency in the mesenteric lymph nodes (Fig 3B), comparable to that seen in the draining popliteal and inguinal lymph nodes of mice immunized by the SC route (Fig 3C). Likewise, IV and IP administration of SmCB1 did not induce a significant increase in T_H_1 cells in the spleen (Fig 3D), but did increase the frequency of T_H_1 cells in the mesenteric (Fig 3E) and popliteal/inguinal nodes (Fig 3F) when administered by the IP and SC routes, respectively. Lastly, active SmCB1 also induced T_H_17 cells—no induction was apparent in the spleen after IV and IP administration (Fig 3G), but IP and SC administration induced significant increases in T_H_17 cell frequency in the mesenteric (Fig 3H) and popliteal/inguinal nodes (Fig 3I), respectively. Together these findings suggest that active SmCB1 induces a pleiotropic CD4^+^ T cell response, consisting of T_H_1, T_H_2 and T_H_17 components, by multiple routes of administration (IP and SC).

### SmCB1 proteolytic activity is required for the induction of T_H_1, T_H_2 and T_H_17 cells

**Fig 4:**
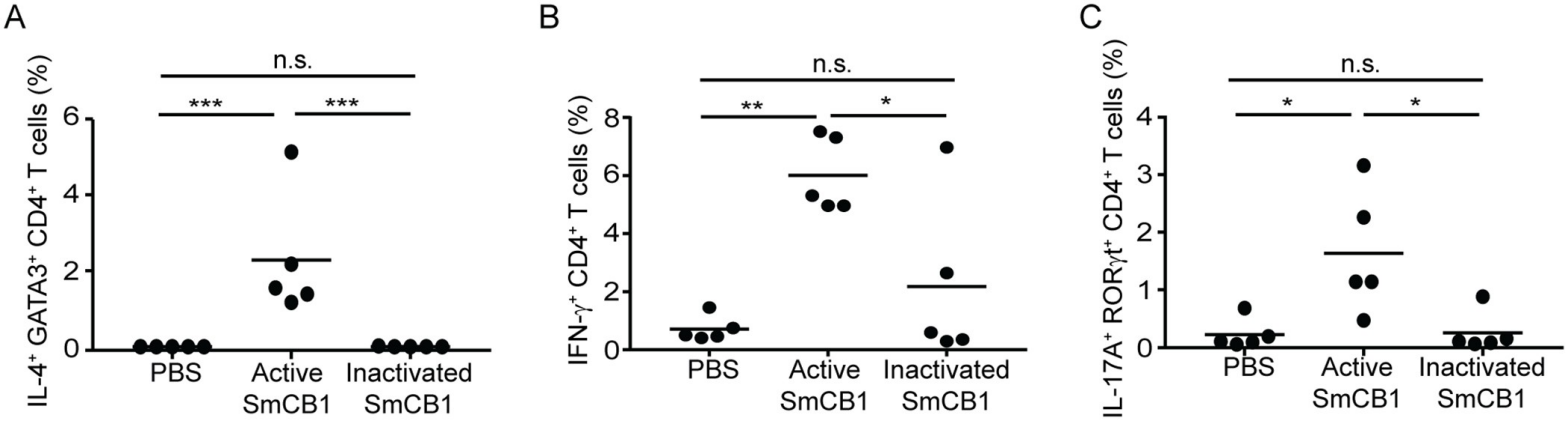
The capacity of SmCB1 to induce T_H_2, T_H_1 and T_H_17 cells is dependent on its proteolytic activity. On day 22, intracellular cytokine/transcription factor staining and flow cytometry were used to determine the frequencies of IL4^+^ GATA3^+^ CD4^+^ T cells (A), IFN-γ^+^ CD4^+^ T cells (B), and IL-17A^+^ RORγt^+^ CD4^+^ T cells (C), in the mesenteric lymph nodes of wild type C57BL/6 mice that received injections of PBS, active SmCB1, or inactivated SmCB1, on days 0, 14 and 21 by the intraperitoneal route. n.s., *P* > 0.05; *, *P* < 0.05; **, *P* < 0.01; ***, *P* < 0.001.

Because IP administration of active SmCB1 induced robust CD4^+^ T cell responses in the mesenteric nodes, which yield significantly more cells than the popliteal and inguinal nodes, we used this route of administration to test whether the catalytic activity of SmCB1 was required for T_H_ cell induction. Either active or E-64-inactivated SmCB1 was administered to separate groups of C57BL/6J mice on day 0, 14 and 21 by IP injection (25 μg/mouse), and the T_H_ cell frequencies in the mesenteric nodes the day after the last injection were compared with those in mice that received PBS alone. Catalytic inactivation of SmCB1 significantly reduced the induction of T_H_2 (Fig 4A), T_H_1 (Fig 4B) and T_H_17 cells (Fig 4C) compared to active SmCB1, and in the case of T_H_2 and T_H_17 cells, inactivated SmCB1 appeared to have no stimulatory capacity at all (Fig 4A and 4C). These data suggest that the ability of SmCB1 to induced T_H_1, T_H_2 and T_H_17 cells is dependent on the enzyme’s proteolytic activity.

### Acute schistosome infection induces a transient T_H_17 response

The finding that active SmCB1 induced a robust T_H_17 response led us to hypothesize that acute schistosome infection may also induce a hitherto unappreciated T_H_17 response. To test this hypothesis, we infected groups of wild type C57BL/6J mice with *S*. *mansoni* by percutaneous exposure to cercariae, and then evaluated the frequency of T_H_1, T_H_2 and T_H_17 cells in the spleens and mesenteric lymph nodes at 4, 6 and 8 weeks post infection. Consistent with our previous findings [20], the frequency of IL-4^+^ CD4^+^ T cells was significantly increased at all time points in the spleen (S3 Fig, part A) and mesenteric nodes (S3 Fig, part B), with the frequency increasing as the infection progressed. In the spleen, elevated frequencies of GATA3^+^ CD4^+^ T cells were detected at 6 and 8 weeks post infection (S3 Fig, part C), and at all three time points in the mesenteric nodes (S3 Fig, part D), with frequencies again increasing as the infection progressed. Using the most stringent definition of both IL-4 and GATA3 positivity, increased frequencies of T_H_2 cells were detected in the spleen at week 8 (S3 Fig, part E) and at all three time points in the mesenteric nodes (S3 Fig, part F).

For T_H_1 cells, elevated frequencies were detected at 6 and 8 weeks post infection in both spleen (S3 Fig, part G) and mesenteric nodes (S3 Fig, part H), with frequencies reduced at week 8 compared to week 6, consistent with the down-modulation of the T_H_1 response that occurs at the onset of oviposition [18].

**Fig 5:**
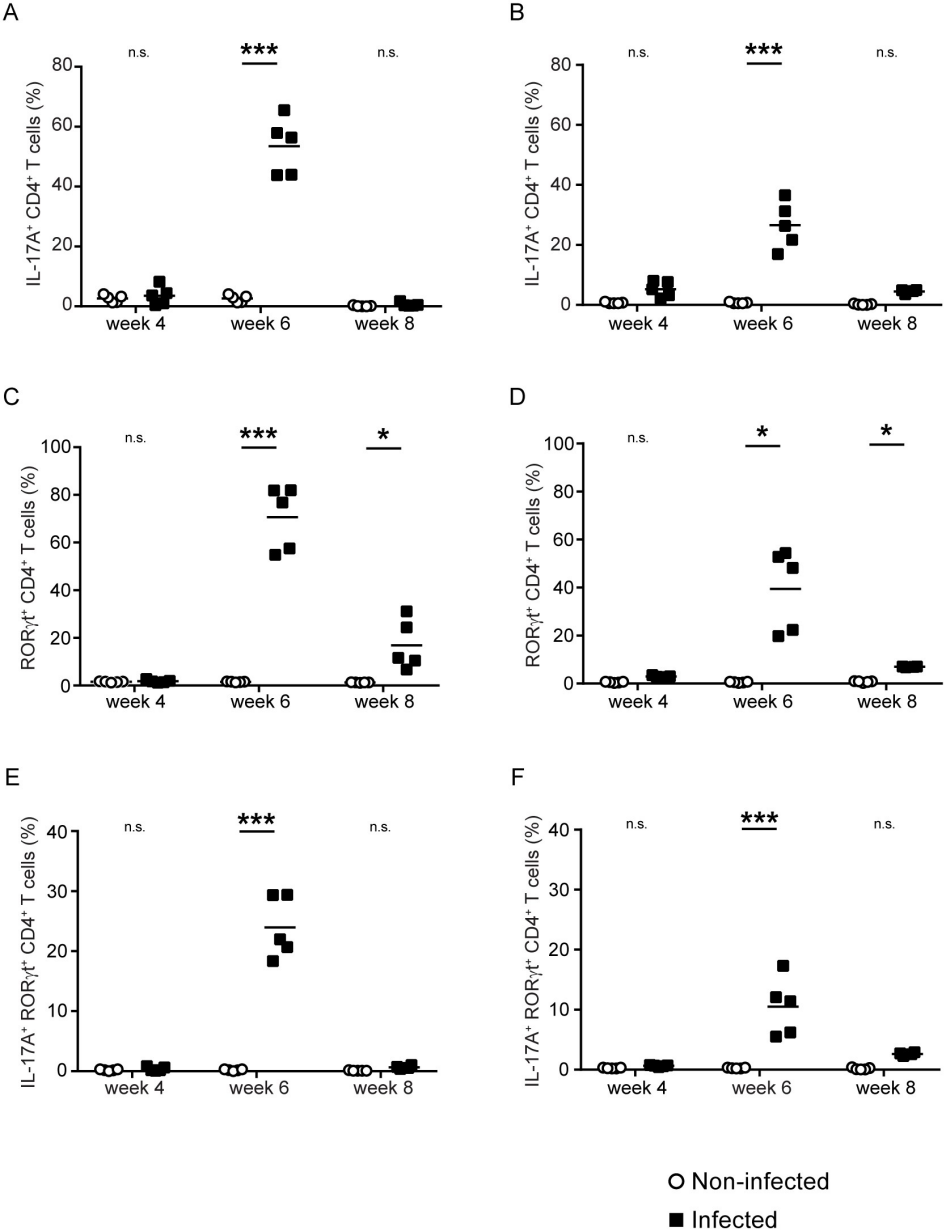
Acute schistosome infection induces a transient T_H_17 response in the spleen and mesenteric lymph nodes. Using intracellular cytokine/transcription factor staining and flow cytometry, the frequency of IL-17A^+^ CD4^+^ T cells (A, B), RORγt^+^ CD4^+^ T cells (C-D), and double-positive IL-17A^+^ RORγt^+^ CD4^+^ T cells (E, F) in the spleens (A, C, E) and mesenteric lymph nodes (B, D, F) of *S*. *mansoni*-infected C57BL/6 mice, was determined at 4, 6 and 8 weeks post infection. Non-infected mice were included at each time point, as negative controls. n.s., *P* > 0.05; *, *P* < 0.05; ***, *P* < 0.001.

For T_H_17 cells, significantly elevated frequencies of IL-17A^+^ CD4^+^ T cells were detected in the spleen (Fig 5A) and mesenteric nodes (Fig 5B) at week 6, but not at week 4 or 8. Significantly elevated frequencies of RORγt^+^ CD4^+^ T cells were detected in spleen (Fig 5C) and mesenteric nodes (Fig 5D) at 6 and 8 weeks post infection, with frequencies at 8 weeks substantially decreased relative to week 6. Using the most stringent definition of both IL-17A and RORγt positivity, frequencies of T_H_17 cells were elevated in spleen (Fig 5E) and mesenteric nodes (Fig 5F) at week 6 only. These data suggest that acute schistosome infection induces a transient T_H_17 response that, like the T_H_1 response, is down-regulated at the onset of oviposition.

### T_H_17 cells induced by schistosome infection are responsive to schistosome antigens

**Fig 6:**
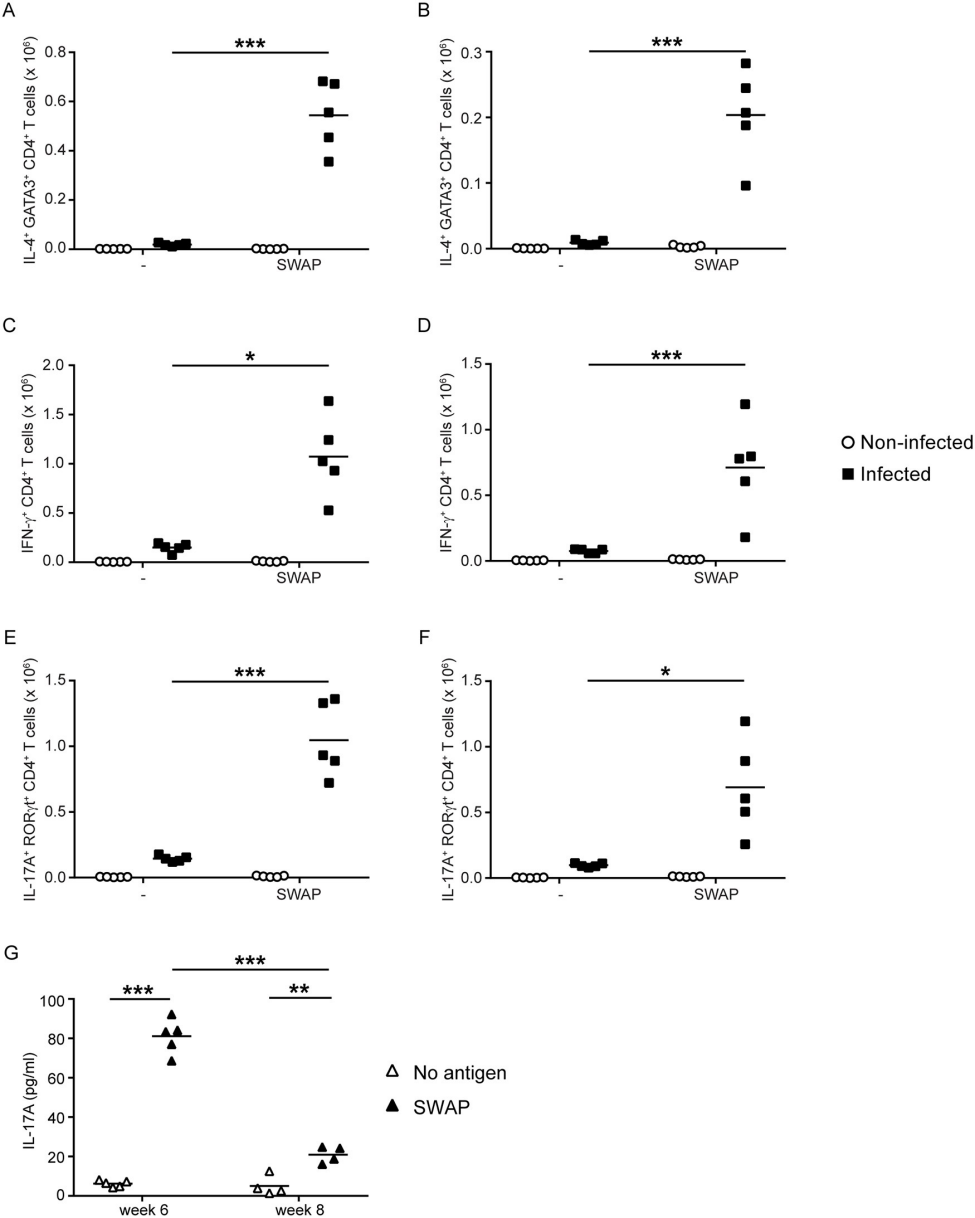
T_H_2, T_H_1 and T_H_17 cells induced by acute schistosome infection proliferate and produce cytokine in response to schistosome worm antigens. At 6 weeks post infection, cells from the spleens (A, C, E) and mesenteric lymph nodes (B, D, F) of *S*. *mansoni*-infected mice were cultured *in vitro* in the absence (-) or presence of *S*. *mansoni* worm antigens (SWAP). Spleen and mesenteric lymph node cells from non-infected mice were included as controls and cultured under identical conditions. After three days in culture, intracellular cytokine/transcription factor staining and flow cytometry were used to determine the number of IL4^+^ GATA3^+^ CD4^+^ T cells (A, B), IFN-γ^+^ CD4^+^ T cells (C, D), and IL-17A^+^ RORγt^+^ CD4^+^ T cells (E, F) in the cultures. G, spleen cells from mice that were infected with *S*. *mansoni* for 6 and 8 weeks were cultured *in vitro* either in the presence or absence of *S*. *mansoni* worm antigens (SWAP). After three days of culture, the concentration of IL-17A in the culture supernatant was determined by ELISA. *, *P* < 0.05; **, *P* < 0.01; ***, *P* < 0.001.

To test whether the T_H_17 cells induced by schistosome infection were specific for and responsive to schistosome antigens, spleen and mesenteric lymph node cells from non-infected mice and mice infected for 6 weeks with *S*. *mansoni* were cultured *in vitro* with SWAP for 3 days. At the end of the culture period, the numbers of T_H_1, T_H_2 and T_H_17 cells in the cultures were compared to cultures where the antigen was omitted. The numbers of T_H_2 cells expanded significantly in response to SWAP in both spleen (Fig 6A) and mesenteric node (Fig 6B). Likewise, T_H_1 cells expanded significantly in response to SWAP stimulation, in both spleen (Fig 6C) and mesenteric node (Fig 6D). Finally, T_H_17 cells from the spleen (Fig 6E) and the mesenteric nodes (Fig 6F) also expanded in numbers in response to SWAP. These data suggest that, like the T_H_1 and T_H_2 cells induced during schistosome infection, the T_H_17 cells induced by acute schistosome infection are specific for schistosome worm antigens.

Lastly, splenocytes from mice that were infected with *S*. *mansoni* for 6 and 8 weeks were cultured *in vitro* for 3 days either with or without SWAP, and the accumulation of IL-17A in the supernatant was measured by ELISA. We found that SWAP stimulated significant secretion of IL-17A by splenocytes at 6 and 8 weeks post infection (Fig 6G), with the 6 week splenocytes producing more IL-17A than the 8 week cells. These data further support the conclusion that the T_H_17 cells induced by acute schistosome infection are specific for schistosome worm antigens and that this response is subject to down-modulation by 8 weeks post infection.

### Cytokine transcript abundance mirrors the frequency of T_H_ phenotypes during acute schistosome infection

**Fig 7:**
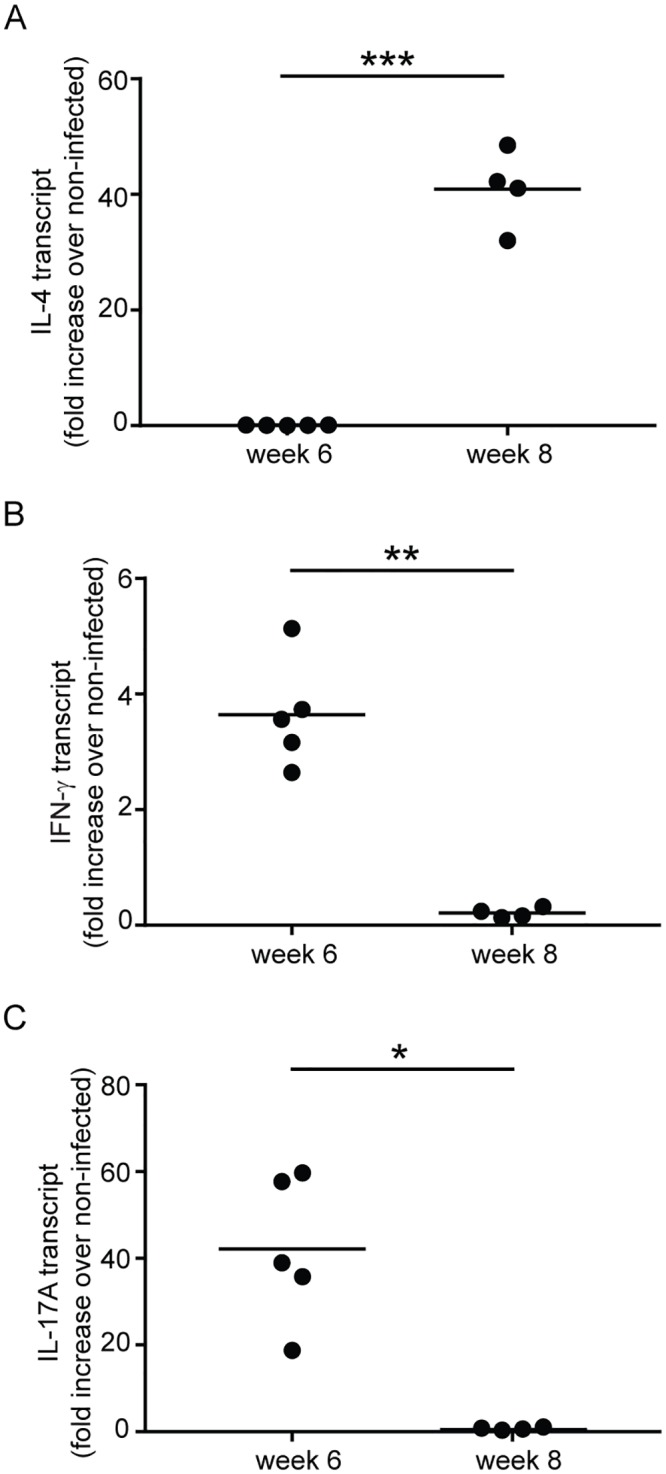
The abundance of IL-4, IFN-γ and IL-17A mRNA mirrors the relative frequency of T_H_2, T_H_1 and T_H_17 cells at 6 and 8 weeks post infection with *S*. *mansoni*. The relative abundance of IL-4 (A), IFN-γ (B) and IL-17A (C) mRNA in spleen cells from *S*. *mansoni*-infected mice at 6 and 8 weeks post infection was determined by quantitative real-time PCR. Results are expressed as fold change in the experimental groups compared to equivalent cells from non-infected control mice. *, *P* < 0.05; **, *P* < 0.01; ***, *P* < 0.001.

To corroborate our flow cytometry data on the relative abundance of T_H_1, T_H_2 and T_H_17 cells using another independent methodology, we estimated the relative abundance of IL-4, IFN-γ and IL-17A transcripts in RNA extracted from splenocytes at 6 and 8 weeks post infection. IL-4 transcript was most abundant at week 8 (Fig 7A), while IFN-γ (Fig 7B) and IL-17A transcripts (Fig 7C) were most abundant at week 6, consistent with the conclusion that the T_H_1 and T_H_17 responses induced by acute schistosome infection are down-modulated after the onset of oviposition.

## Discussion

Because proteolytic activity is considered to be essential to the T_H_2-promoting capacity of proteases [30], we attempted to test the contribution of SmCB1 activity to the induction of T_H_2 responses during the first four weeks of schistosome infection. Previously, *in vivo* administration of the cysteine protease inhibitor, K11777, to schistosome-infected mice was found to be highly effective at ablating SmCB1 activity [38]. We therefore investigated pre-patent CD4^+^ T cell responses in infected mice under K11777 treatment. Our finding that both T_H_2 and T_H_1 responses were significantly reduced by K11777 treatment could be interpreted as evidence that SmCB1 activity contributes broadly to initiation of CD4^+^ T cell responses during schistosome infection and not specifically to T_H_2 cell induction. However, this interpretation is confounded by the potential off-target effects that K11777 treatment may have. For example, we cannot rule out the possibility that other schistosome proteases with potential immunological roles, such as *S*. *mansoni* cathepsins L and E [34], were not also inhibited. Also, host lysosomal cysteine proteases are important in the presentation of antigens by antigen-presenting cells (APCs) to CD4^+^ T cells via the major histocompatibility complex (MHC) class II pathway, being required both for antigenic peptide processing and for degradation of the invariant chain [43,44]. Interference by K11777 with APC protease activity could therefore have potentially broad effects on CD4^+^ T cell responses. Finally, despite specifically selecting doses of K11777 that do not interfere with parasite establishment or alter gross parasite morphology, we cannot rule out the possibility that K11777 had some effect on schistosome development or antigen expression and thus altered parasite immunogenicity. Given these caveats, we focused instead on testing the immunological significance of SmCB1 activity by comparing the immunogenicity of active and chemically inactivated recombinant SmCB1.

The native proteolytic activity of SmCB1 was previously shown to induce protection against challenge infection with cercariae, as irreversible inhibition of SmCB1 significantly diminished, though did not ablate, the protection conveyed by SmCB1 immunization [37]. Consistent with these results, we found that SmCB1 had robust T_H_2-inducing capacity that was dependent on the enzyme’s protease activity. T_H_2 cell induction by SmCB1 was independent of the detection method employed (IL-4 reporter mice, intracellular cytokine and transcription factor staining), the genetic background of the mice (BALB/c, C57BL/6), and the route of SmCB1 administration (subcutaneous, intraperitoneal). Interestingly, no cytokine responses were detected to SmCB1 administered intravenously. We hypothesize that, when administered by this route, perhaps SmCB1 is rapidly inactivated by circulating protease inhibitors such as α-2-macroglobulin [45], before any immunological effects can be exerted.

The ability to elicit CD4^+^ T cell responses in the mesenteric lymph nodes by intraperitoneal administration of SmCB1 afforded the opportunity to use intracellular cytokine and transcription factor staining and flow cytometry to detect other effector phenotypes among the responding CD4^+^ T cells. Unexpectedly, we found that active SmCB1, but not inactivated SmCB1, also elicited robust T_H_1 and T_H_17 responses, in addition to T_H_2 cells. While we were not able to detect traces of endotoxin in our SmCB1 preparations, we considered the possibility that the immunostimulatory activity was due to contaminating pattern recognition receptor (PPR) ligands of microbial origin. However, if this were the case, we would not expect the immunostimulatory capacity of SmCB1 to have been sensitive to catalytic inactivation. Furthermore, previous studies showed that, in mice immunized with SmCB1 and subsequently infected with cercariae, SmCB1 elicited IFN-γ and IL-17 production from cells in the lymph node draining the site of infection 4 days after challenge [36], suggesting that the protease may have broad CD4^+^ T cell-priming activity that extends beyond T_H_2 cell induction. How SmCB1 proteolytic activity simultaneously induces T_H_1, T_H_2 and T_H_17 responses is an open question, but perhaps the most parsimonious explanation is that, in this instance, each of the three effector phenotypes shares a common initiator mechanism. In support of this argument, we note that PARs have been implicated in the induction of T_H_1 [46] and T_H_17 [47] responses, as well as T_H_2 responses [28–30]. Alternatively, SmCB1-mediated proteolytic damage to cells and the extracellular matrix may mobilize a broad repertoire of alarmins and endogenous damage-associated molecular patterns (DAMPs) [48]. In addition to the IL-33, IL-25 and TSLP that promote T_H_2 cell differentiation by activating ILC2s [33], other DAMPs may activate dendritic cells and other APCs to produce the IL-12 required for T_H_1 induction [49], and the IL-1β, IL-6 and IL-23 that promote T_H_17 differentiation [50,51].

T_H_1 responses induced by schistosome worm antigens are an acknowledged feature of pre-patent schistosome infection, detectable within the first four weeks of infection and subsequently down-modulated at the onset of parasite egg-laying [10,18,20]. In contrast, T_H_17 responses have been primarily detected later in infection, driven by egg antigens, and in mouse strains prone to severe egg-induced pathology (reviewed in [52]). Our finding that active SmCB1, a worm antigen, stimulated a T_H_17 response prompted us to investigate whether T_H_17 responses are induced during acute schistosome infection. Our analysis of T_H_1, T_H_2 and T_H_17 cell frequency from 4 to 8 weeks post infection, in a strain not considered to be prone to pathogenic T_H_17 responses (C57BL/6) [52], revealed a transient T_H_17 response that peaked at 6 weeks post infection and was subsequently down-modulated. The timing of this induction and down-regulation resembles that of the pre-patent T_H_1 response, which is down-regulated by egg-induced T_H_2 responses [6,53], suggesting that the T_H_17 response we observed may be regulated by similar mechanisms. Failure to induce a T_H_2 response results in a persistent T_H_1 response and excessive inflammation and pathology [6,54]. If the T_H_17 response we observed is controlled by similar regulatory mechanisms, a persistent T_H_17 response could also contribute to the pathology and morbidity that accompanies a failure of the T_H_2 response [6]. T_H_2 responses have been shown to limit IL-17-mediated pathology in other models of helminth infection [55]. There is also a precedence from other models of T_H_2-mediated inflammation, e.g. allergic airway disease [56], for T_H_17-mediated pathology to emerge when T_H_2 responses are impaired.

Among inbred mouse strains, certain strains (e.g. CBA, C3H) are considered “high pathology” strains with regard to schistosome infection, as these animals develop more severe pathology upon infection, characterized by large, poorly circumscribed granulomas around parasite eggs that are driven by T_H_17 responses [57–59]. Imbalances in IL-1β and IL-23 production by dendritic cells in response to schistosome egg antigens were found to underlie the preferential development of T_H_17 responses in CBA mice [57,58] suggesting that fundamental differences in the dendritic cell response to egg antigens distinguishes high pathology strains from other mouse strains. One question arising from these findings is whether high-pathology strains also differ in their response to worm antigens during acute infection, and whether persistent T_H_17 responses to worm antigens contribute to pathology in these strains. Since there is evidence that immune responses to worm antigens during pre-patent infection can prime subsequent responses to eggs after the onset of oviposition, presumably due to the expression of common antigens and antigenic cross-reactivity between life cycle stages [60], persistent T_H_17 responses may set the stage for a subsequent pathogenic T_H_17 response to schistosome eggs.

Gene expression analyses that compared dendritic cells from high-pathology CBA mice and low-pathology C57BL/6 mice revealed significant differences in the expression of C-type lectin receptors (CLRs) between the two strains, with CBA dendritic cells expressing 18-fold higher levels of CD209a (SIGNR5) compared to C57BL/6 dendritic cells [61]. Furthermore, ablation of CD209a expression in CBA dendritic cells, and overexpression of CD209a in C57BL/6 dendritic cells, revealed that CD209a was required for the expression of IL-1β and IL-23 by egg-stimulated dendritic cells and for the subsequent development of T_H_17 cells [61]. Recently, it was shown that signaling through three CLRs, CD209a, Dectin-2 and Mincle, together augment the production of IL-1β and IL-23 by egg-stimulated dendritic cells, and is required for T_H_17 development and immunopathology [62]. These findings firmly implicate carbohydrate antigens, rather than proteases, in the induction of T_H_17 responses to schistosome eggs, and suggest that multiple different schistosome antigens, from different life cycle stages, might contribute to T_H_17 induction throughout infection. Indeed, although SmCB1 may contribute to T_H_17 induction, there may well be other worm antigens also driving T_H_17 polarization during pre-patent infection. We note that secretory-excretory products of schistosome worms, which contains SmCB1 but also many other antigens, also induce T_H_17 responses when used to immunize mice [63], suggesting that other worm antigens may contribute to this response.

We considered the possibility that the sharp increase in frequency of T_H_17 cells at week 6 post infection, in spleen and mesenteric lymph node, was due to bacterial translocation from the intestine and exposure to microbial PRR ligands [64]. However, the timing of the T_H_17 response suggests it is induced before the onset of parasite egg-laying and before significant egg-induced damage of the intestinal mucosa would occur. Furthermore, the T_H_17 cells present at 6 weeks post infection are clearly specific for and responsive to worm antigens, suggesting they are induced by the schistosome infection and not by microbial antigens.

In summary, our data suggest that a secreted parasite cathepsin is a broadly stimulatory immunogen with capacity to initiate T_H_17 as well as T_H_1 and T_H_2 responses. This led to the finding that acute schistosome infection in a low-pathology mouse strain is associated with a transient T_H_17 response to worm antigens that is rapidly down-regulated at the onset of egg production. Understanding the development and regulation of T_H_17 responses in models of schistosome infection will be relevant to elucidating the mechanisms that underlie the development of pathology in human schistosomiasis, which remain poorly understood. One recent study revealed a positive correlation between the frequency of circulating T_H_17 cells and the occurrence of bladder pathology in *S*. *haematobium*-infected children [65]. Furthermore, significantly elevated levels of IL-17 are detectable in the plasma of symptomatic acute schistosomiasis patients (T.A. Pereira, personal communication). Associations between pathology and T_H_17 responses have also been noted in other human helminth infections [66] and animal models of helminthic disease [67,68]. Our findings provide new insights into the role of T_H_17 responses in the natural history of schistosomiasis and suggest these potentially pathogenic responses warrant further investigation, both in animal models and naturally infected human subjects.

## Supporting information

S1 Fig Gating strategy to identify IL-4-GFP^+^ CD4^+^ T cells by flow cytometry. Top row, left to right: a FSC-A vs. FSC-H plot is used to gate on singlet events (left panel); a FSC-A vs. SSC-A plot is used to gate on lymphocytes (center panel); a CD19 histogram is used to exclude CD19^+^ cells (right panel). Bottom row, left to right: a CD4 vs. CD3 plot is used to gate on double-positive CD3^+^ CD4^+^ cells in the upper right (Q2) quadrant (left panel); a TCRβ vs. CD4 plot is used to gate on double-positive TCRβ^+^ CD4^+^ cells in the upper right (Q2) quadrant (middle panel); a GFP vs. TCRβ plot is used to identify IL-4-GFP^+^ cells in the upper right (Q2) quadrant (right panel).
(EPS)

S2 Fig Representative flow cytometry data demonstrating the enumeration of T_H_2, T_H_1 and T_H_17 cells induced by immunization with SmCB1. On day 22, intracellular cytokine/transcription factor staining and flow cytometry were used to determine the frequencies of IL4^+^ GATA3^+^ CD4^+^ T cells (A), IFN-γ^+^ CD4^+^ T cells (B), and IL-17A^+^ RORγt^+^ CD4^+^ T cells (C), in the spleens, mesenteric lymph nodes, and popliteal and inguinal lymph nodes of wild type C57BL/6 mice that had received injections of active SmCB1 on days 0, 14 and 21, by intravenous (IV), intraperitoneal (IP) and subcutaneous (SC) routes. Cells from comparable tissues of animals that received no antigen (-) were included as negative controls.
(TIF)

S3 Fig Dynamics of T_H_2 and T_H_1 cell frequency during acute schistosome infection. The frequency of IL-4^+^ CD4^+^ T cells (A, B), GATA3^+^ CD4^+^ T cells (C, D), double-positive IL-4^+^ GATA3^+^ CD4^+^ T cells (E, F), and IFN-γ^+^ CD4^+^ T cells (G, H), in the spleens (A, C, E, G) and mesenteric lymph nodes (B, D, F, H), of *S*. *mansoni*-infected C57BL/6 mice at 4, 6 and 8 weeks post infection, and in non-infected mice, were compared using intracellular cytokine/transcription factor staining and flow cytometry. n.s., *P* > 0.05; *, *P* < 0.05; **, *P* < 0.01; ***, *P* < 0.001.
(EPS)

S1 Table List of antibodies used for flow cytometry. Information regarding the source and format of all antibodies used in the flow cytometric analysis of immune cells is provided.
(XLSX)
