# Supplementary figures and images for: A secreted schistosome cathepsin B1 cysteine protease and acute schistosome infection induce a transient T helper 17 response
Source: PLoS Negl Trop Dis. 2019 Jan 17;13(1):e0007070. doi: 10.1371/journal.pntd.0007070 (PMC6353221; doi:10.1371/journal.pntd.0007070)

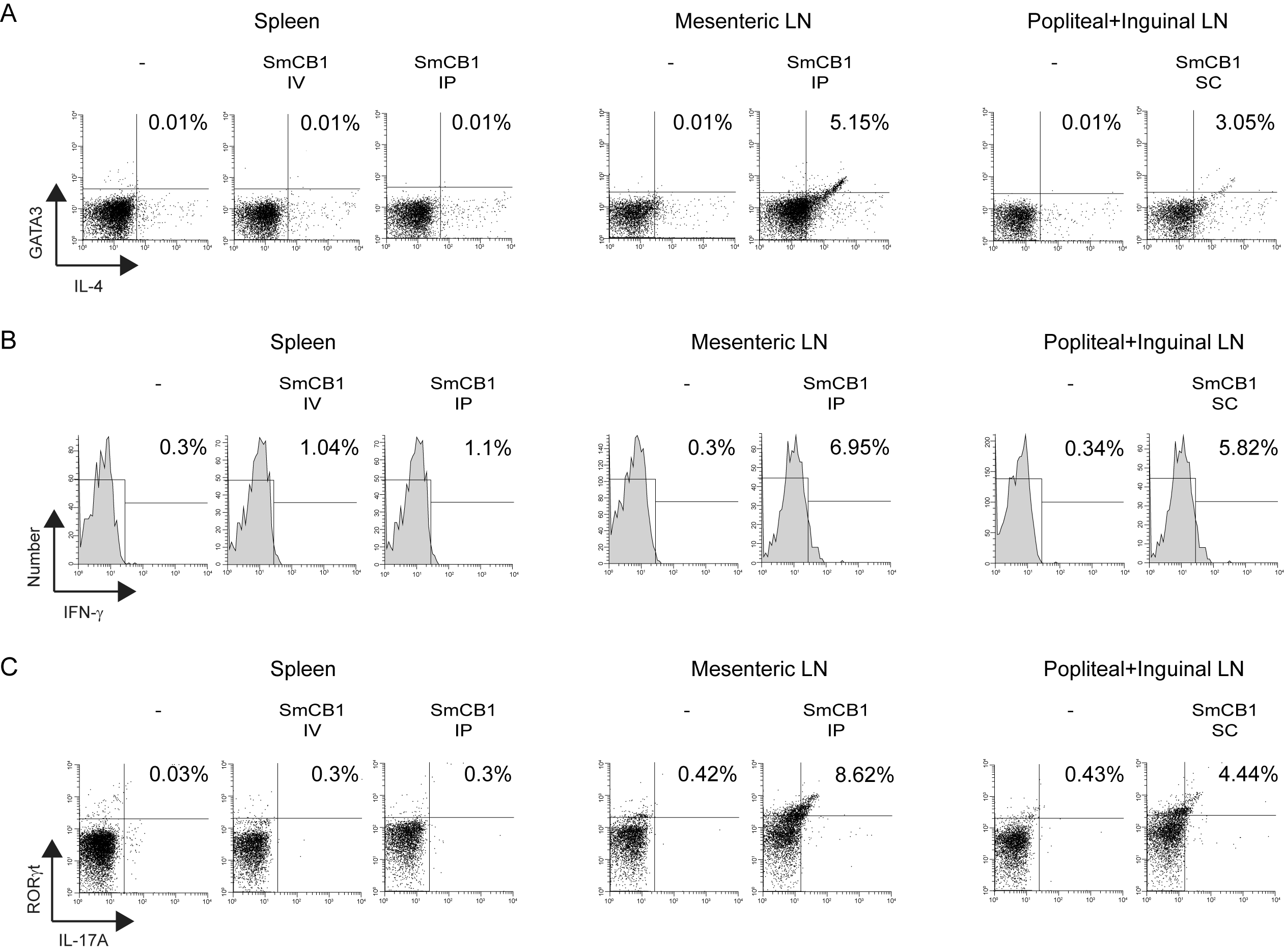

Supplement: S2 Fig — On day 22, intracellular cytokine/transcription factor staining and flow cytometry were used to determine the frequencies of IL4+ GATA3+ CD4+ T cells (A), IFN-γ+ CD4+ T cells (B), and IL-17A+ RORγt+ CD4+ T cells (C), in the spleens, mesenteric lymph nodes, and popliteal and inguinal lymph nodes of wild type C57BL/6 mice that had received injections of active SmCB1 on days 0, 14 and 21, by intravenous (IV), intraperitoneal (IP) and subcutaneous (SC) routes. Cells from comparable tissues of animals that received no antigen (-) were included as negative controls. (TIF) [file pntd.0007070.s002.tif]
